# Biotechnological Approaches to Producing Natural Antioxidants: Anti-Ageing and Skin Longevity Prospects

**DOI:** 10.3390/ijms24021397

**Published:** 2023-01-11

**Authors:** Sarah Bouzroud, Ezzouhra El Maaiden, Mansour Sobeh, Nawal Merghoub, Hassan Boukcim, Lamfeddal Kouisni, Youssef El Kharrassi

**Affiliations:** 1 African Sustainable Agriculture Research Institute (ASARI), Mohammed VI Polytechnic University (UM6P), Laâyoune 70000, Morocco; 2 AgroBioSciences Department (AgBS), Mohammed VI Polytechnic University (UM6P), Benguerir 43150, Morocco; 3 Green Biotechnology Center, Moroccan Foundation for Advanced Science, Innovation & Research (MAScIR), Rabat 10100, Morocco

**Keywords:** antioxidants, anti-ageing, biotechnology, callus, plant tissue culture, skin

## Abstract

Plants are the main source of bioactive compounds that can be used for the formulation of cosmetic products. Plant extracts have numerous proven health benefits, among which are anti-ageing and skin-care properties. However, with the increased demand for plant-derived cosmetic products, there is a crucial prerequisite for establishing alternative approaches to conventional methods to ensure sufficient biomass for sustainable production. Plant tissue culture techniques, such as in vitro root cultures, micropropagation, or callogenesis, offer the possibility to produce considerable amounts of bioactive compounds independent of external factors that may influence their production. This production can also be significantly increased with the implementation of other biotechnological approaches such as elicitation, metabolic engineering, precursor and/or nutrient feeding, immobilization, and permeabilization. This work aimed to evaluate the potential of biotechnological tools for producing bioactive compounds, with a focus on bioactive compounds with anti-ageing properties, which can be used for the development of green-label cosmeceutical products. In addition, some examples demonstrating the use of plant tissue culture techniques to produce high-value bioactive ingredients for cosmeceutical applications are also addressed, showing the importance of these tools and approaches for the sustainable production of plant-derived cosmetic products.

## 1. Introduction

The global cosmetic industry is experiencing rapid growth due to the increase in beauty awareness and the growing interest in beauty care, skin protection, and cosmetic products. With the expansion of this lucrative sector, a new trend has emerged among consumers for natural, sustainable, and green-derived cosmetic products instead of synthetic ones, resulting in an increase in the global plant extracts market [1]. For instance, the size of this market was valued at USD 10.19 billion in 2021 and is expected to increase exponentially to USD 22.49 billion in 2030 [2,3]. This great interest is justified by the outstanding therapeutic properties of plant extracts with no or few adverse effects [4].

Plants have always been a major source of bioactive ingredients with a broad spectrum of uses. They have proven health and wellness benefits including providing antioxidants and protection against UV radiation, as well as containing anti-wrinkle, anti-cancer, anti-ageing, and skin-altering (whitening, moisturizing, or soothing) properties [5]. However, due to the large demand for plant-derived cosmetic products, nowadays, several plant species are being over-exploited (harvested in great numbers from their natural habitats) or endangered. In addition, challenges, such as slow growth, seasonal harvests, the presence of toxic compounds in their chemical composition, and variations in bioactive compounds both among plants and from one harvest to another, can be limiting factors for their potential use in the cosmetic industry [4]. These limitations can be overcome with the application of plant tissue culture techniques combined with biotechnological tools and approaches.

Plant tissue culture techniques deliver an appealing alternative for producing bioactive compounds. They can easily be defined as biotechnological techniques that rely on the use of nutritive culture media and controlled aseptic conditions to ensure the culture and development of plant cells, tissues, and organs [6]. This technology shows many advantages over conventional agricultural practices such as (i) secondary metabolite production that occurs independent of the intrinsic or extrinsic factors that may affect crop growth and yield, (ii) the yield of desired phytochemical constituents by ensuring a constant production, and (iii) production under controlled conditions, thus limiting the risk of contamination by agrochemicals (pesticides, insecticides, herbicides, chemical fertilizers) [7].

To date, many green-label products (plant-derived products) have been developed using plant tissue culture techniques. Some of them have applications in cosmetics, more precisely, for the treatment of skin ageing and skin disorders occurring as a result of skin exposure to external agents [5,8]. Therefore, the foremost objective of this comprehensive review is to provide a concise overview of the broad applications of plant tissue culture techniques and biotechnological approaches to the production of high-value bioactive compounds that can be used in the formulation of cosmetic products, with a focus on skin disorder prevention and skin anti-ageing molecules. The review also covers some examples of cosmetic formulations that have been patented during the last decade in which plant tissue culture techniques were applied to produce the active molecules in those formulations.

## 2. Skin Ageing: Significance and Causes

### 2.1. Skin Anatomical Structure and Key Functions

**Figure 1:**
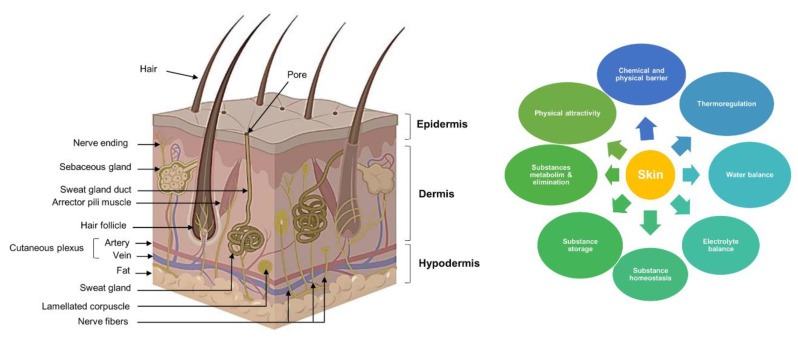
Skin anatomical structure (with three distinct layers: epidermis, dermis, and hypodermis) and key roles (the skin anatomical structure schema was adopted from Kobayashi et al., (2020) and Nielson et al., (2021) [13,14].

Skin is the prime organ in the human body and represents one-sixth of the total weight of the human body. The skin is anatomically formed with three main layers: the epidermis, dermis, and hypodermis, also known as the subcutaneous tissue (Figure 1) [9]. The external layer (epidermis) comprises five distinct layers: a basal layer (stratum basal), spinous layer (stratum spinosum), granular layer (stratum granulosum), clear layer (stratum lucidum), and horny layer (stratum corneum) [10]. The dermis, which is the median layer, is intricately connected to the epidermis through the basement membrane. Unlike the epidermis, which is made up of different cellular layers tissues, the dermis primarily comprises the extracellular matrix (ECM), an acellular component. This acellular matrix is mainly formed by collagen fibers, which account for 75% of the skin’s dry weight and offer plastic strength and elasticity [11]. Besides collagen, the ECM proteins include elastin and glycoproteins, which comprise sweat pores and hair follicles and harbor the lymphatic, vascular, and neuronal systems [9]. The third layer, the hypodermis, contains fat cells (adipocytes) rearranged in fat lobules, which form loose connective tissue [12].

Skin plays a wide range of functions. It offers chemical and physical protection for the body against external injuries such as pathogens, UV radiation, chemical threats, dehydration, and temperature fluctuations [8]. It also ensures water and electrolyte balance (sweat glands and epidermal barrier) and thermoregulation (thermoreceptors) and participates in the immune response (skin-associated lymphoid tissues (SALT)). The presence of nerve endings, such as Meissner, Pacinian, and Ruffini corpuscles, along with Krause’s bulbs and Merkel cells, confers a sensory function to the skin. Another important function is linked to the homeostasis, metabolism, storage, selective absorption, and elimination of substances [12]. Besides these biological functions, skin plays a role in physical appeal (Figure 1) [9].

### 2.2. Skin Ageing

Skin ageing is one of the most common dermatological concerns affecting human skin. It is commonly characterized by the loss of elasticity and skin tone, the emergence of fine wrinkles and age spots, and the failure to repair wounds. Skin ageing is a multi-factorial process driven by both internal (genetic background, hormones, time, etc.) and external (pollution, UV radiation exposure, etc.) factors [15]. It is biologically characterized by epidermal and dermal-epidermal junction changes, along with an overall degradation of the dermal extracellular matrix. The latter is mostly caused by alterations to collagen and elastin fibers [16]. Skin ageing can appear because of time (chronological ageing resulting from intrinsic factors) or photoaeging (photoaged skin due to extrinsic factors). Chronological ageing causes a reduction in the dermal and epidermal layers, a decrease in the number of nerve endings, and the production of sex hormones, which lead to the loss of sensitivity. This skin-ageing process occurs mostly due to the over-accumulation of Reactive Oxygen Species (ROS). In general, it is established that 1.5–5% of oxygen consumed by cells is transformed by internal processes into ROS [8]. ROS can be produced in the mitochondria (keratinocytes and fibroblasts), endoplasmic reticulum (cytochrome P450), and cytosol (ROS produced by arachidonic acid metabolism) [17]. ROS production can be triggered by the overexpression of interleukins. Besides the increase in ROS production, skin ageing is also linked to the high and low expressions of matrix metalloproteinases (MMPs) and MMP inhibitors (TIMP), respectively. The variations in the expression of these specific proteinases are proven to be associated with the variation in the amount of ROS. The increased amounts of ROS lead to the upregulation of the activator protein-1 (AP-1), which, in turn, prompts collagen degradation by MMP and triggers the activation of nuclear factor kappa B (NF-κB) and a set of inflammatory responses [8].

Skin ageing is more pronounced in photoaged skin with deeper wrinkles, a more noticeable loss of elasticity, and a coarse skin texture [8]. Various cellular stressors are involved in this physiological process such as (i) alterations to chromatin organization, (ii) telomere shortening, (iii) mitochondrial dysfunction, (iv) oncogene activation (v) epigenetic strands, (vi) inflammation, and (vii) immunosuppression [15,18]. Oxidative stress induced by UVR is the main cause of photoageing. Skin exposure to UV radiation induces the activation of the neuroendocrine system. This activation occurs either by chemical mediators or through nerve transmission [8]. In addition, the hypothalamic–pituitary–adrenal (HPA) axis is also activated by photoageing, leading to the induction of homeostatic responses to alleviate the damage. UV-R-induced DNA damage occurs due to ROS imbalance, which leads to the production of a specific marker of DNA oxidative impairment, 8-hydroxy-2′-deoxyguanosine (8-OH-dG) [19]. In addition, other markers, such as interleukin-6 (IL-6), tumour necrosis factor α (TNF-α), and cyclooxygenase enzymes, are also activated by ROS. At the gene expression level, ROS accumulation triggers NF-κB expression and the expression of inducible nitric oxide synthase (iNOS), which facilitates hyperpermeability and angiogenesis by upregulating the vascular endothelial growth factor (VEGF) [20]. Besides NF-κB, mitogen-activated protein kinases (MAPKs) are also activated, thereby increasing MMP gene transcription. The upregulation of MMP genes negatively affects ECM homeostasis and triggers elastin and collagen degradation [8].

## 3. Plant-Derived Bioactive Compounds with Anti-Ageing Properties

Plant-derived bioactive compounds (PDBCs) stemming from plant metabolism generally display antioxidant, anti-inflammatory, and antibacterial properties and also have promising anti-ageing properties [21,22]. Commonly known as secondary metabolites, PDBCs are chemical elements that are not directly involved in plants’ normal growth processes [21]. They are usually produced in response to environmental constraints as a defence against predators, pathogens, or UV radiation; for interspecific competition between species; or to aid in reproduction (pheromones, coloring agents, and attractive smells) [23]. These natural compounds can be divided into vitamin (vitamins C and E), polyphenol (flavonoids, phenolic acids, stilbenes, and lignans), and terpenoid groups [24].

### 3.1. The Main Production Routes for Plant-Derived Bioactive Compounds

Plant-derived bioactive compounds display significant biological activities. For centuries, these high-value molecules have been extracted from plants harvested from nature. However, given the low yield of production of these molecules, several alternatives have emerged with the development of synthetic chemistry, the discovery of microbial and fungal engineering (heterologous production), and the development of plant tissue culture techniques. The commercial supply of PDBCs is usually a major problem associated with the compounds’ availability. For instance, it is broadly admitted that the occurrence of secondary metabolites is often limited to less than 1% of the dry weight of the plant [25,26]. Thus, natural harvest is usually impractical since it cannot supply sufficient amounts of the desired compound [27]. For instance, an average of 1 kg of blueberries is required to produce 38 mg of quercetin-3-glucoside [28]. Natural harvest can also be limited by the seasonal availability of plant material, its growth and development rates, and species abundance in nature [29]. Besides the low production yield of bioactive compounds obtained from natural harvest, the production of these valuable compounds by plants is not usually satisfactory since their production can, in some cases, be induced only under specific conditions or during a plant-specific development stage [30,31].

Chemical synthesis can also be proposed as an alternative approach to producing some bioactive compounds such as carotenoids. The chemical synthesis of astaxanthin costs an average of 1000 USD/kg, which is more than the cost of the natural harvest of *Haematococcus pluvialis* [32,33]. This alternative production route shows, however, the issues associated with the strict conditions of the chemical reactions involved and the production of undesirable side products and by-products [34,35].

Another production pathway has emerged with the discovery and development of microbial and fungal biotechnology. This technology, also called heterologous production, mostly relies on the use of microorganisms such as yeast, fungus, or bacteria. Through this process, several molecules, among which are resveratrol, a secondary metabolite known for its great medicinal and cosmetic potential (anti-ageing), were successfully produced in high amounts using genetically engineered microbial hosts, such as *Corybacterium glutamicum*, *Escherichia coli,* and *Lactococcus lactis,* or recombinant yeasts such as *Saccharomyces cerevisiae* [36,37,38,39]. Resveratrol is produced by plants in variable amounts, such as 0.36 mg/kg from *Vitis vinifera* fruits, 1 mg/kg from *Ampelopsis japonica* roots, and 7.95 mg/kg from *Morus alba* aerial parts [40], but remains lower than that reported using recombinant microorganisms. For example, resveratrol production can reach 812 mg/L or 2.34 g/L using recombinant yeast or bacteria, respectively [37,41]. Note that the success of heterologous production is dependent on the knowledge and understanding of the biosynthetic pathways of the desired bioactive compounds, even though the molecular cascade involved in the biosynthesis of several bioactive compounds has already been characterized. However, the major limitations of this technology are linked to the low activity of plant-derived enzymes in the microbial hosts (yeast, bacteria, or fungus), along with the low precursor supply. Thus, further studies should be conducted to identify the best candidate enzymes and the optimal conditions that allow for the conversion of the metabolic intermediates into the desired products, which will avoid the accumulation of intermediate molecules in the microbial hosts [35].

Besides heterologous production, plant in vitro culture techniques have been proposed as a sustainable alternative for producing plant-derived bioactive compounds. These techniques offer a series of advantages compared to the use of whole plants, mainly (i) pathogen removal, (ii) the scaling-up of production through the use of a bioreactor once the process of cultivation is well mastered, which can efficiently reduce operational costs, and (iii) the easy purification and processing of natural products [42,43]. Thus, on this basis, plant tissue culture techniques have recently been used to produce bioactive ingredients. Several examples have underlined the efficiency of these techniques such as the production of taxol, podophyllotoxin, phytoene, or berberine from *Taxus* sp., *Podophyllum hexandrum*, *Citrus sinensis,* and *Coptis japonica* cell cultures, respectively [44,45,46,47].

### 3.2. Dermo-Protective Action of Plant-Derived Antioxidants

Photoprotective products derived from PDBCs have gained increasing attention in recent decades as they can prevent oxidative stress, UVA radiation damage, and skin cancer. Natural antioxidants, including polyphenols, flavonoids, vitamin C, and vitamin E, have recently been used in sunscreen and the formulation of cosmetics due to their high antioxidant properties, which can form a protective barrier against UV radiation. Table 1 and Table 2 summarize the most pertinent research that has been carried out on human and murine cell lines, respectively, to study the dermo-protective effects of essential oils, plant extracts, and extracts derived from plant tissue cultures.

## 4. Plant Tissue Culture Techniques for the Production of Valuable Antioxidants

Plant in vitro cultures can be used as natural sources of antioxidants [25]. Using these culture systems, antioxidant molecules can be produced in high amounts independent of the environmental conditions. Several in vitro approaches can be used such as micropropagation, in vitro root cultures (hairy roots), or callus and cell suspensions.

### 4.1. Adventitious Roots and Hairy Roots

It is well established that in vivo roots produce, in most cases, more phytochemical substances than other plant organs. On this basis, an in vitro root-culture-based technology has been developed for the production of pharmaceutical secondary metabolites [76]. In vitro adventitious roots provide the ability for metabolite production for an extended period of time in plant in vitro culture systems without losing genetic stability. This outstanding property is conferred by the highly differentiated nature of in vitro root cultures [77]. Two different root cultures can be distinguished: untransformed and transformed hairy root cultures. Untransformed roots can be obtained through explant cultures in the presence of specific phytohormones, which are more likely to be auxins such as indole-butyric acid (IBA), indole-3-acetic acid (IAA), or naphthalene acetic acid (NAA) alone, or in some cases are combined with cytokinins [78]. Unlike untransformed root cultures, transformed cultures are developed through the inoculation of plant explants with *Agrobacterium rhizogenes*. Transformed hairy roots have been obtained from many monocots and dicotyledonous plant species. The success of this method is mostly attributed to the T-DNA region in the Ri plasmid of *Agrobacterium*, which plays a crucial role in the effective introduction of foreign or external DNA into the nuclear genome of the host plant cell [77]. The infection process was found to be linked to the existence of *rol* genes in the T-DNA region. The *rol* genes comprise the *rolA*, *rolB,* and *rolC* genes and account for the induction of secondary metabolite biosynthesis, with *rolB* being the most powerful among the three genes [79]. The transformation technology offers the possibility of introducing foreign gene encoding for therapeutic plant secondary metabolite pathway enzymes into plant cells to heighten the production of a specific metabolite [77,79]. Some research has reported the success of this technology in producing molecules of interest. Rosmarinic acid production was successfully achieved using transformed hairy roots obtained the from shoot culture of *Salvia officinalis* transfected with *Agrobacterium rhizogenes* A4 and ARCC 15834 strains [80]. Rosmarinic acid was also produced from elite hairy roots obtained by the *Agrobacterium rhizogene*-mediated transformation of *Ocimum basilicum* and *Dracocephalum moldavica* plants [81,82]. The rosmarinic acid amount produced was slightly variable within species, ranging between 76.41 mg/g of DW obtained using the *Ocimum basilicum* hairy root culture and 78.05 mg/g of DW recorded using the *Dracocephalum moldavica A. rhizogene*-transformed roots [81,82]. Rosmarinic acid production was previously investigated by Shekarchi et al. (2012) in 21 plant species belonging to the Labiatae family that were grown in nature [83]. The authors showed that the rosmarinic acid content ranged between 0 and 58.8 mg/g of DW for different species, which was lower than the values obtained using transformed hairy roots. The production yield using this in vitro culture system was higher than that produced using in vivo-cultivated roots, which provides a viable alternative for the scale-up production of high-value compounds.

It is widely established that chemical compounds secreted by roots represent a reservoir of defense molecules used by plants to enhance their natural defense mechanisms and protect themselves against pathogenic attacks [84]. Many hairy root cultures have drawn noteworthy attention due to their high-value phytochemical compounds such as resveratrol from *Arachis hypogaea*, indole alkaloids from *Catharanthus roseus*, artemisinin from *Artemisia annua,* and camptothecin from *Camptotheca acuminata* or *Ophiorrhiza pumila* [85]. Sena et al. (2018) reported the presence of glucosinolates, hydroxycinnamic acids, and their glucoside derivatives in *Brassica rapa* hairy root cultures. The hydro-alcoholic-derived extract displayed a potent anti-melanogenic activity, strong inhibition of intracellular tyrosinase activity, and significant reduction in melanin content in primary melanocytes. This anti-melanogenic activity was comparable to the inhibition potential of the well-known synthetic tyrosinase-inhibiting drug, kojic acid [71]. In addition, the glucoproteic preparation obtained from the same *Brassica rapa* hairy root cultures showed similar hypopigmented activity and reduced melanin accumulation through the negative regulation of tyrosinase activity. It also interfered with the signal transduction cascade of melanogenesis and regulated the expression of the microphthalmia-associated transcription factor, which is known as a key regulator of melanin synthesis [71]. *Panax ginseng* hairy root cultures over-expressing the *PAP1* gene (production of anthocyanin pigment 1) showed increased production of polyphenolic compounds including anthocyanin [85]. Moreover, the hydro-alcoholic extracts obtained from the transformed hairy root cultures showed outstanding dermo-protective properties, with strong inhibitory action against elastase and tyrosinase enzymes [70,86].

### 4.2. In Vitro Propagation

In vitro propagation, or micropropagation, is a variant of the vegetative mode of propagation accomplished using plant-derived explants cultivated under aseptic in vitro conditions [87]. It offers the possibility of producing a large number of plants that can be explored for the extraction of valuable metabolites while reducing the over-exploitation of wild and endangered species [88]. The use of differentiated plantlets (micropropagated plants) is mandatory when the bioactive molecule is exclusively produced in specialized plant organs or tissues (e.g., essential oils). Another advantage of the use of in vitro-propagated plants is associated with their stability and higher yields of secondary metabolites. The use of in vitro culture systems allows for production independent of seasonal constraints and the rapid and efficient isolation of the targeted bioactive molecule, along with the reliability and predictability of production [25].

Some studies have pointed out the efficiency of in vitro propagation in terms of the production of bioactive compounds. Goyali et al. (2013) found that lowbush blueberry clones obtained through micropropagation displayed higher flavonoid and phenolic contents compared to those developed using the conventional method of propagation [89]. Similar findings were reported in *Ziziphora tenuior* L. by Dakah et al. (2014) [90]. The authors found that in vitro-propagated plant extracts of *Ziziphora tenuior* L. showed higher radical scavenging ability than wild plants. They also explained this noticeable difference by the stress conditions that arise through the in vitro culture establishment or the presence of plant growth regulators, which can have a stimulatory effect on polyphenol production [90]. *Heperzia serrata*, an important traditional herb in Chinese culture, is known to produce a valuable compound, Huperzine A (HupA). *Heperzia serrata* extracts obtained from micropropagated plants displayed increased antioxidant activity. However, the production of HupA remains lower in micropropagated plants than in wild ones. Hypericin content subsequently increased in *Hypericum hookerianum* micropropagated plant extracts in comparison with the extract generated from wild plants [91]. In the in vitro culture of *Salvia officinalis*, abietane diterpene was only detected in shoot cultures but not in cell suspensions, calluses, or hairy roots [92]. Despite the above, in some cases, the phenolic composition and antioxidant activity can be lower in micropropagated plants compared to wild ones, such as in *Cichorium pumilum* Jacq [93], *Caralluma tuberculata* [94], and *Alocasia longiloba* Miq [95].

### 4.3. Callogenesis and Cell Suspensions

Plants show noteworthy developmental plasticity for cell differentiation, as it is the main feature of plant cells. Due to this outstanding property, plants can form unorganized cell masses, referred to as calluses, in response to environmental constraints, most likely pathogen invasion or physical damage [96]. Callus culture establishment relies mostly on the dedifferentiation of cells. This can be defined as the process by which mature or specialized cells lose their differentiated character and become juvenile (dedifferentiated) [97].

Through their transfer to a liquid medium, callus culture clumps can desegregate into small pieces, aggregates, or even single cells, whereby cell suspension cultures are achieved. A callus is typically heterogeneous. Cell suspensions are a potential source of high-value plant-derived bioactive compounds [97,98]. Cell suspensions encompass a homogeneous cell population, which produces uniform and rapid nutrient and plant growth regulators. They also easily accommodate several biotechnological strategies such as elicitation, precursor feeding, and bioconversion or biotransformation, as well as mass production in bioreactors (scale-up) [7]. Several important plant-derived bioactive compounds have been produced using callogenesis and cell suspension technologies, whereby the majority were obtained using cell suspensions [98]. The main PDBCs that have been produced using cell suspensions are Echinan 4 P, Acetos 10 P, Teoside 10, and Teupol 50 P [99,100].

Many studies have reported the efficacy of cell suspensions for producing desired bioactive compounds. For example, ginsenoside production was obtained through *Panax quinquefolium* cell suspensions developed in an MS medium in the presence of 1 mg/L of 2.4-Dichlorphenoxyacetic acid and 0.25 mg/L of Kinetin [101]. Shikonine production was assessed from cell suspensions of *Onosma bublotrichum* in an MS medium supplemented with 0.2 mg/L of IAA and 2.10 mg/L of Kinetin for calluses and in an SH medium for cell suspensions [102]. *Glycyrrhiza uralensis* cell suspensions were able to produce significant amounts of flavonoids in a Murashige and Skoog medium supplemented with a combination of 2,4-D, NAA, and BA and elicited with methyl jasmonate [103]. 20-hydroxyecdysone was obtained from both *Achtranthes bidentata* and *Vitex glabrata* cell suspensions grown in the presence of both NAA and 0.2 mg/L of BA for *Achtranthes bidentata* and the presence of 2,4-D and BA for *Vitex glabrata* [104].

Several previous studies have underlined the great potential of callus and cell cultures in the treatment of skin disorders. *Dilochos biflorus* stem cell culture-derived hydrosoluble extract was characterized by Bilmonte et al. (2014) for its high amount of isoflavones, mainly daidzein, genistin, and their glucosidic derivatives. The authors found that the generated extract showed a noticeable inhibitive action of UV-induced erythema, which highlighted the protective effects of these plant-derived compounds against UV radiation, specifically against sunburn and solar erythema [105]. Later, Imparato et al. (2016) used skin artificial models to demonstrate the UV protection capacity of *Dilochos biflorus* cell culture extracts on ECM components [106]. This outstanding dermo-protective activity was linked to the extract’s ability to scavenge free radicals, inhibit collagenase production on the dermis, and preserve collagen structure for up to 72 h after UVA radiation exposure [106]. Butterfly bush (*Buddleja davidii*) extracts obtained using cell suspension cultures produced high amounts of verbascoside, a phenylpropanoid glycoside compound known for its versatile protective properties (antioxidant, chelator, anti-inflammatory). Exploring the dermatological properties of the generated extracts showed the strong skin repair capacity and skin inflammation preventative action of this extract, attributed to the strong inhibition of collagenase activity and the repression of pro-inflammatory factors [107]. Bengal coffee (*Coffea bengalensis*) plant cell culture extracts are caffeine-free and displayed great potential for use in skin care. For instance, it was shown that the hydrosoluble extract derived from *Coffea bengalensis* cell cultures prompted collagen I and II synthesis in fibroblasts, promoted lipase activity, and stimulated the expression of hydration-related genes in the keratinocytes [108].

## 5. Main Biotechnological Approaches to Increasing the Production of Plant-Derived Bioactive Compounds

**Figure 2:**
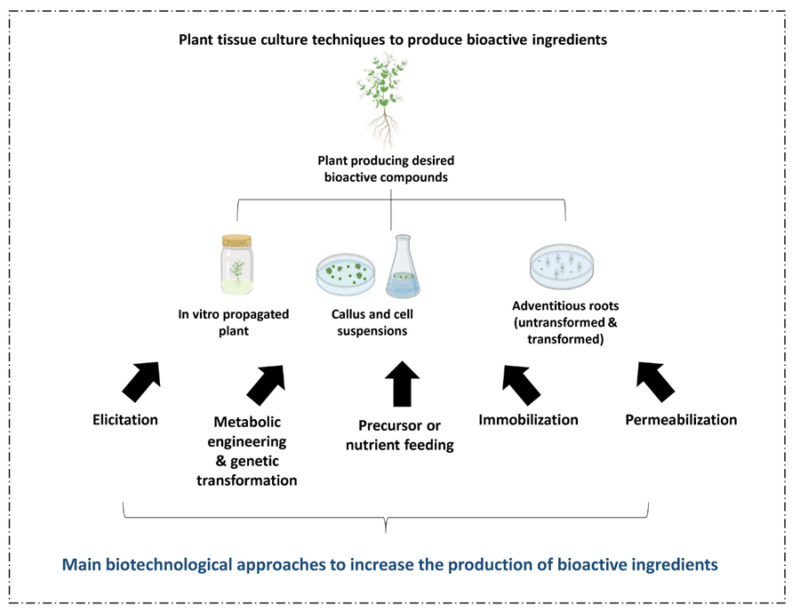
Main biotechnological approaches available for enhancing the production of bioactive compounds using plant cell culture systems.

Plant cell and tissue culture (PCTC) provides a promising biotechnological tool for generating a broad number of phytochemicals for pharmaceutical purposes. However, only some successful cases are available on the market due to minimal phytochemical productivity, which is insufficient to cover the culture costs [76]. Thus, during the last decade, research has been oriented towards enhancing the production of high-value phytochemicals without increasing the production costs to scale up the use of in vitro culture techniques as “chemical factories” [109]. Several strategies, among which are elicitation, metabolic engineering, immobilization, permeabilization, and two-phase systems, have been broadly used to increase the production of PDBCs (Figure 2) [77].

### 5.1. Elicitation

Elicitation is one the most efficient procedures applied nowadays to improve the biotechnological production of PDBCs. Elicitation requires the use of specific compounds, commonly known as elicitors, to prompt plant defense and trigger secondary metabolite biosynthesis and production [110]. Two distinct types of elicitors can be distinguished: abiotic and biotic elicitors. Abiotic elicitors gather all non-biological substances, such as inorganic compounds, for example, metal ions or salts (calcium chloride, silver nitrate, magnesium sulfate, mercury chloride, cobalt chloride, zinc ions, etc.), known to stimulate the production of bioactive substances through their plant secondary metabolism adjustment [43]. Unlike abiotic elicitors, biotic elicitors have a biological origin. They are either used as crude extracts or partially purified pathogen or plant-derived products. They can be either of a complex composition, such as fungus and yeast extracts, or a specific composition such as glycoproteins, purified chitosan, alginate, xanthan, polysaccharides, etc. [111]. Several parameters, among which are the elicitor type, concentration, time of exposure, culture type, medium composition, cell line, stage, and age of the culture, are the main factors affecting the efficiency of the elicitation procedure in PDBC production [112].

Elicitation has been widely used to increase PDBC production in in vitro cultures. Several reports have underlined the efficiency of this method. The elicitation of *Pueraria candollei* suspension cells using salicylic acid enhanced isoflavonoid production and accumulation, more specifically, khwakhurin, daidzein, puerarin, and genistin, which are molecules that display great anti-ageing properties [57]. In *Solanum xanthocarpum*, callus culture elicitation using blue light resulted in a peak production of methyl-caffeate, esculetin, caffeic acid, and scopoletin. These molecules are known for their great antioxidant, anti-inflammatory, antidiabetic, and anti-ageing activity [113]. The NaCl-induced salt stress application to a cultivated cardoon (*Cynara cardunculus* L. var *altilis*) callus increased the total phenolic and antioxidant content, which resulted in an increase in pro-collagen and aquaporin production in dermal cells, thereby boosting the production of bioactive compounds that can be used for cosmetic formulations [114]. Methyl jasmonate elicitation applied to *Isatis indigotica* hairy root cultures demonstrated outstanding results in the production of lignans. It also allowed for the discovery of AP2/ERFs TFs that have been implicated in the production of this class of bioactive compounds, as well as upregulated biosynthetic genes, which underlines the importance of eliciting in the identification of key regulatory mechanisms that can be used for metabolic engineering in in vitro cultures [115]. Other examples of the efficacy of eliciting the stimulation of PDBC production are shown in Table 3.

### 5.2. Precursor and Nutrient Feeding

Precursor feeding is a biotechnological strategy that depends on the ability of plants and plant cell cultures to convert precursors (supplemented by the media culture) into desired products using pre-existing enzymes [135,136]. This technology has been broadly employed to trigger the production of specific compounds. For instance, numerous reports have demonstrated the efficiency of precursor feeding in the stimulation of PDBC synthesis. *Linum album* hairy root cultures fed with a known lignan precursor, coniferaldehyde, resulted in a considerable increase in pinoresinol, lariciresinol, and podophyllotoxin production [137]. In *Centella asiatica* leaf-derived callus and cell suspensions, asiaticoside accumulation was achieved by the addition of amino acids to the culture media, more precisely, leucine [138]. Karppinen et al. (2007) reported similar findings for adhyperforin production from *Hypericum perforatum* shoot cultures. For instance, the authors found that the administration of isoleucine and valine to the shoot culture was responsible for the production of hyperforin. By tracking the insertion of isoleucine and valine using labelled forms of these amino acids, the authors discovered that these two amino acids were incorporated into the acyl side chain of both adhyperforin and hyperforin [138].

Following the same principle as precursor feeding, nutrient feeding aims to increase the yield of PDBCs by adjusting the physical and chemical factors of the culture media. This strategy was proven to be effective for biomass enhancement and the production of ginsenoside from ginseng adventitious root cultures. In fact, as reported in [139], biomass production and ginsenoside amount increased when the culture was replenished with a freshly prepared culture medium. Similar findings were also reported for the production of caffeic by-products from *Echinacea purpurea* adventitious root cultures [140] and taxol production from *Taxus chinensis* cell suspensions [141].

### 5.3. Metabolic Engineering

Metabolic engineering is defined as the production of specific substances or molecules, such as pharmaceuticals, chemicals, fuels, and drugs, by disrupting the metabolic pathways in cells [142]. It gives a brand-new standpoint to better understand the PDBC biosynthesis routes through overexpression studies. It can also imply the repression of other pathways (competitive pathways) to enhance the metabolic flow of the specific biosynthesis route mediators to ensure elevated production [143]. The main objective of this strategy is to prompt cellular activity through the manipulation of cell functions using recombinant DNA technology. So far, several strategies, such as the introduction of genes isolated from the same species or different organisms, promoters enhancing target gene expression (constitutive expression of targeted genes using 35S promoter, for example), or disruptive expression of targeted gene or genes (antisens, RNA interference, or CRISPR/Cas9 technologies), have been used to achieve this purpose [144]. The most common example of genetic manipulation is the use of *Agrobacterium tumefasciens*-mediated genetic transformation, which can allow for the introduction of the desired gene.

The genetic disruption of biosynthesis pathway intermediates can also be conducted using other alternative transformation methods such as protoplast transformation, biolistics (microprojectile bombardment), liposome-mediated transformation, or pollen-tube pathways [143]. Metabolic engineering offers many advantages for the scale-up production of bioactive compounds by over-expressing genes (responsible for the production of regulatory enzymes) that are involved in their biosynthetic pathways [145]. However, given the complexity of the regulatory process in plant cells and the presence of critical and rate-limiting enzymes responsible for the feedback regulation of the abundance of bioactive compounds, the production of PDBCs through metabolic engineering is limited. Thus, additional investigations are required to identify the rate-limiting steps and their regulation [146,147].

### 5.4. Immobilization

Immobilization is one of the key strategies that can be applied to enhance the production of PDBCs in PCTC systems. It relies on the use of a gel matrix that allows cell entrapment. At the same time, cells are exposed to high concentrations of ions to neutralize the undesirable impact on cell metabolism. This strategy has attracted scientists and researchers worldwide as it allows for the increase in cell viability and stability of the bioactive compounds produced, in addition to the increase in the production of desirable molecules [148]. For cell entrapment or immobilization, several chemicals, such as agarose, alginate, agar, and polyacrylamide combined with alginate, can be used as the gel matrix. Alginate polymers are the most common substances used for cell immobilization, as they show the best results in terms of PDBC production yields. For instance, *Eurycoma longifolia* cell aggregate entrapment with 2.5% of alginate polymer for three weeks resulted in a substantial rise in the production of 4H-imidazol-4-one, canthin-6-one, and strictosidine-synthase compared to non-immobilized cells [149]. For chitosanase production from *Gongronella* sp. cells, the highest production was achieved using cell immobilization with calcium alginate (E404) gel combined with polyurethane foam at pH 5.5 [150]. In *Juniperus chinensis*, Premjet et al. (2007) found that the production of podophyllotoxin increased by 96–98% in entrapped cells using an alginate polymer [151]. *Plumbago rosea*-immobilized cells using E404 resulted in a threefold increase in the production of plumbagin, the important bioactive compound reported in this plant species, compared to non-entrapped cells [152,153]. The beneficial effects of cell immobilization can be explained by the fact that the gel (polymer) matrix generates an appropriate diffusion gradient over the immobilized cells, which improves biochemical communication. Polymer matrices automatically trigger the establishment of cell aggregates, thereby reducing cells’ dependence on the culture media, resulting in a higher yield of PDBCs [148]. Although cell immobilization increases PDBC production, bioactive compounds are often entrapped and frequently stored within cell vacuoles. Thus, the cell immobilization and production process are economically dependent on the cell’s capacity to secrete the desirable bioactive compounds into the adjacent medium, which can occur naturally using natural (passive and active transport) or artificial (permeabilization strategy) secretion mechanisms [135].

### 5.5. Permeabilization

As mentioned above, PDBCs are usually entrapped in specialized organs or cell structures, usually in cell vacuoles. Hence, PDBC release into the culture medium coupled with an appropriate purification procedure can allow for the recuperation of desired compounds. The permeabilization strategy relies on the use of chemical or physical approaches to increase the permeability of plant cell membranes. The chemical-mediated permeabilization can easily be implemented using organic solvents, such as dimethylsulfoxide [DMSO] and isopropanol, and polysaccharides such as chitosan [135]. For taxol, hexadecane, dibutylphthalate, or decanol were used to increase *Taxus chinensis* cell culture permeability in [141]. Other permeabilization methods, such as electric fields and sonication, can be applied to recover PDBCs from cell vacuoles [135]. Note that the accumulation of PDBCs can be altered either by the feedback regulation (inhibition) of product synthesis or by the degradation of bioactive compounds in the media. This obstacle can be avoided by using in situ product removal, which involves a direct liquid–liquid or liquid–solid separation [154], where the latter showed better results than the liquid–liquid culture system. For solid–liquid systems, XAD4, XAD7 resins, and activated charcoal are commonly used. For instance, it was previously demonstrated that the use of XAD7 improved the production of ajmalicine and serpentine in *C. roseus*, plumbagin in *Pitytriasis rosea*, an alkaloid in *Eschscholzia californica,* and taxuyunnanine C in *Taxus chinensis* [155,156,157,158]. XAD4 was successfully applied to the production of anthraquinones from *Morinda elliptica* [159].

## 6. Production of Antioxidant Substances for Cosmetic Formulations Using Biotechnology

PCTC techniques in combination with different biotechnological approaches aiming to produce high amounts of PDBCs have led to the development of several cosmetic products with anti-ageing and dermo-protective activity. Some of them have been patented, from which several cosmetic products have been developed by leading companies in the cosmetic industry. Below are some examples of patents that have been registered in the last decade. They were randomly selected to show concrete applications of biotechnology, mainly plant tissue culture techniques, to the formulation of galenic and cosmetic products:

- A patent registered in the United States by Blum et al. in 2012, related to the development of dedifferentiated plant cells from *Malus domestica* cv Uttwiler Spaetlauber fruits and their use in the formulation of cosmetic preparations to ensure the protection of stem cells against both internal and external stress factors, promotion of stem cell proliferation, and prevention of cell apoptosis (Patent US 8,580,320 B2). From these cell suspensions, different cosmetic preparations have been developed, among which are vanishing creams, liquid balms, intensive hair masks, and eye creams. The efficiency of the developed cosmetic preparations has been tested on stem cells originating from umbilical cords, hair follicles, and fibroblasts.
- *Syringa vulgaris* plant cells were successfully generated from the in vitro culture of plant tissues under aseptic conditions in growth containers supplemented with specific plant growth regulators by an Italian team (Dal Monte et al., 2006; Patent number: US 7,718,199 B2). An aqueous extraction was performed on callus-derived cell suspensions. HPLC profiling revealed the presence of significant amounts of verbascoside and isoverbascoside. The cell-suspension-derived extracts showed strong antioxidant and scavenging activity against free radicals. Moreover, the developed extracts displayed great anti-hair-loss properties due to their capacity to inhibit 5-alpha reductase and lipoxygenase. The generated extracts also showed strong anti-tyrosinase activity and notable skin-whitening properties.
- Undifferentiated cells of *Iris* plants (*Iris pallida*, *Iris germanica,* and *Iris florentina*) were generated by Breton and Gueniche in 2001. Galenic preparations were developed from the generated cells. Following the inventors’ claims, the developed preparations included sunscreens, with active ingredients that ensured the protection of the extracellular matrix proteins, such as from UV radiation, through the enzymatic inhibition of MMP proteins (Breton and Gueniche in 2001, Patent number: EP 1 174 120 B1).
- *Leontopodium alpinum* undifferentiated cells obtained using in vitro cell cultures were used for the formulation of cosmetic preparations by French inventors (Gracioso et al.). The discovery was published as a patent by the inventors in 2016 (Patent deposition in 2015, Patent number: WO 2016/113659 A1). The developed product was proposed as a cosmetic treatment for skin-aged cell homeostasis restoration and increasing cell metabolism and energetic activity.
- Undifferentiated cells of *Marrubium vulgare* were used as a raw material for the development of cosmetic preparations by Ringenbach et al. for a well-known cosmetic firm. The patent was registered in 2016. For this patent, a cosmetic composition was prepared from plant cells obtained using the in vitro cell culture process. The inventors proposed this cosmetic preparation for topical treatments to improve skin’s general condition, appearance, and appendages, more precisely, for pore tightening and skin imperfections. From the active ingredient discovered, different galenic formulations have been developed, among which are creams, serums, tissue masks, and cleansing lotions (Patent number: WO 2017/163174 A1).
- A cosmetic formulation was developed by an Italian team (Tito et al.) in 2016. The invention covered by this patent focuses on the use of the somatic embryos of three plant species: *Lotus japonicus*, *Citrus limon,* and *Rosa gardenia*. The generated extracts have shown great action against skin-ageing imperfections and contain skin-tissue rejuvenation properties (Patent number: WO 2016/ 173867 A1).
- A cosmetic product with the ability to protect skin from drying and/or prevent UV-radiation damage was developed from a *Camellia sinensis* var *assamica* dedifferentiated stem cell culture extraction by Berry et al. The developed product was patented in 2017. The invention’s efficiency was tested in human adult dermal fibroblasts. According to the inventors, the generated tea extracts displayed anti-inflammatory properties, prevented skin cell drying, and protected skin cells from UV radiation (Patent number: WO 2017/178238 A1).

## 7. Conclusions

Skin ageing is one of the most frequent dermatological issues affecting human skin and its appearance, resulting in wound repair failure, wrinkle development, and loss of skin tone and elasticity. Several chemical-based products have been developed over the years to prevent skin’s anti-ageing process and reduce its impact. However, with the use of chemical products, several problems have arisen, mostly linked to cell sensitivity, allergies, and the side effects of some chemical products and substances. As an alternative, natural and plant-derived products have been proposed based on their outstanding properties. However, the development of plant-derived bioactive ingredients is highly dependent on plant material, which can be affected by both intrinsic and extrinsic factors. Plant tissue culture techniques can deliver huge quantities of homogenous plant material independent of those factors to ensure the sufficient production of bioactive compounds. In addition, PDBCs can be produced using biotechnological strategies such as elicitation, metabolic engineering, nutrient and precursor feeding, immobilization, and permeabilization. This work presented a comprehensive review of the biotechnological techniques used to produce bioactive compounds, with a focus on antioxidants displaying anti-ageing properties. Some examples of plant tissue culture techniques used in the production of cosmetic products are also addressed to underline the importance of biotechnological tools for the sustainable production of PDBCs.

## Figures and Tables

**Table 1:** Examples of plant-derived bioactive compounds with antioxidant activity that display outstanding skin anti-ageing properties and have been demonstrated in vitro using human cell lines.

Cell Line	Plant Species	Plant Material	Bioactive Molecules (If Applicable)	Noticeable Effects	References
Fibroblasts	*Centella asiatica*	Callus derived from stolon segments	-	* Induction of the expression of antioxidant-encoding genes such as *SOD*, *CAT,* and *GPX*. * Inhibition of the transcription of *MMP-7.*	[48]
	*Chaenomeles japonica*	Callus derived from leaf culture	Triterpenoids	* Stimulation of skin cell proliferation. * Notable antioxidant activity of callus-derived extract.	[49]
	*Cinnamumum cassia*	Bark of in vivo plant material	Trans-cinnamic acid	* Inhibition of intracellular accumulation of ROS. * Downregulation of metalloproteinases (*MMP-1* and *MMP-3*). * Prevention of type I pro-collagen degradation in skin fibroblasts. * Activation of Nrf2 signaling mediated by AMPK, PKC, ROS, or CKII signaling cascades. * Positive regulation of γ-*GCLC* and *HO-1* gene expression because of the activation of Nrf2.	[50]
	*Citrus junos*	Callus derived from leaf, flower, and seed cultures	p-hydroxycinnamoylmalic acid	* Inhibition of melanin biosynthesis. * Strong tyrosinase inhibitory effects. * Enhancement of collagen production. * Acceleration of wound healing process.	[51]
	*Diplectria barbata*	Leaves	-	* A dose-dependent increase in the expression of elastin and collagen genes. * An increase in cell survival, development, and differentiation-related proteins. * Decrease in *MMP-3* expression. * Increase in the expression of *HO-1* and *Nrf2*.	[52]
	*Malus domesticus*	Cell suspensions	-	* Reversal of senescence signs. * Stimulation of the expression of heme oxygenase 1. * Delay in cell apoptosis and senescence.	[53]
	*Olea europaea*	Leaves	-	* Inhibition of cell apoplastic death hypothetically through *TrxR* upregulation. * DNA damage suppression. * Inhibition of ROS production and accumulation.	[54]
	*Panax ginseng*	In vivo plant material	Ginsenoside RG3	* Induction of the expression of extracellular matrix proteins, cell proliferation genes, and genes associated with growth. * Increase in the expression of antioxidant-encoding genes such as *HO-1* and nuclear factor *Nrf2*.	[55]
	*Piper cambodianum*	In vivo plant material	-	* Increase in the expression of extracellular matrix genes such as elastin and collagen and hyaluronan synthase-2 in a dose-dependent manner. * Decrease in the expression of the *MMP-3* gene.	[56]
	*Pueraria candollei*	Cell suspensions derived from stem segment culture	daidzein, genistin, genistein, khwakhurin, daidzin, and puerarin	* Stimulation of cell proliferation. * Strong antioxidant activity estimated through DPPH assay.	[57]
	*Pyrus pyrifolia*	Callus derived from cotyledon culture	-	* Repression of melanin biosynthesis. * Inhibitive action against tyrosinase. * Stimulation of the collagen biosynthesis in fibroblasts. * Acceleration of wound recovery process.	[58]
	*Tiarella polyphylla*	Callus derived from leaf, petiole, and stem segment cultures	Nicotiflorin, astragalin, quercitrin, myricitrin	* Protective effect against UV radiation. * Enhancement of type I pro-collagen amount. * Decrease in MMP-1 production through the repression of the expression of *MMP* genes.	[59]
	*Woodfordia fruticose* Kurz	Callus derived from young apical leaf culture	-	* Collagen and elastin biosynthesis production stimulation. * Positive regulation of the expression of collagen and elastic genes. * Delay in cell senescence.	[60]
Keratinocytes	*Camellia sinensis*	In vivo plant leaves	-	* Accumulation of mRNA and proteins of phase I and II detoxifying enzymes, more likely HO-1. * Positive regulation of Nrf2-mediated pathway by triggering the phosphorylation of ERK and p38. * Downregulation of *MMP-1*.	[61]
	*Lindera erythrocarpa*	Fruit	Lucidone	* Prevention of cell death due to UVA. * Prevention of mitochondria dysfunction, DNA degradation, and Bcl-2/Bax dysregulation. * Induction of antioxidant genes such as *NQO-1*, *HO-A,* and *γ-GCLC* through the transcriptional activation of *Nrf2*. * Reduction of ROS accumulation.	[62]
	*Leontopodium alipnum*	Callus derived from leaf culture	-	* Strong antioxidant activity in response to UV radiation. * Suppression of skin inflammation and wrinkles. * Improvement in skin elasticity, thickness, anti-periorbital wrinkles, and dermal density. * Upregulation of genes involved in cell apoptosis, keratinization, and cornification, forming skin barriers.	[63]
	*Oryza sativa*	Callus derived from seed culture	-	* Strong antioxidant activities of calli extracts. * Increase keratinocyte proliferation. * Strong anti-collagenase and anti-tyrosinase activities recorded.	[64]
	*Phyllanthus emblica* L.	In vivo plant material	-	* Elimination of excessive ROS. * Activation of antioxidant machinery (CAT). * Reduction in the release of cyt c. * Mitigation of the inflammatory mediator PGE2 and the inflammatory signals NF-κB and AP-1. * Inhibition of Akt overactivity.	[65]
	*Polypodium vulgare*	In vivo plant material	Shikimic acid Caffeoylquinic acid derivatives Epicatechin Catechin	* Activation of antioxidant systems. * Dose-dependent regulation of ROS production.	[66]
	*Pyrus pyrifolia*	Callus derived from young leaf culture	Uridine, guanosine, and adenosine	* Strong ABTS and DPPH antioxidant activity. * Notable collagen and elastin glycation inhibitory effect of callus extract. * Stimulation of cell proliferation. * Acceleration of wound healing process. * Stimulation of the production of pro-collagen.	[67]
	*Syzygium samarangense*	In vivo plant leaves	myricetin-3-O-α-rhamnoside 7,8,3′,4′-tetrahydroxy-3,5-dimethoxyflavone	* Reduction in intracellular ROS accumulation and carbonyl amounts. * Maintenance of intracellular GSH levels, even after exposure to toxic chemicals such as sodium arsenite. * Stimulation of Nrf-2 transcription factor translocation to the nucleus, which induces the expression of Mn-*SOD3* and *HO-1* genes. * Inhibition of IκB-α degradation.	[68]
	*Zingiber zerumbet*	Rhizomes	Zerumbone	* Prevention of lactate dehydrogenase release in a dose-proportional way. * Reduction in DNA alteration, ROS synthesis, Bax/Bcl-2 ratio dysregulation, and DNA single-strand breaks. * Transcriptional activation of *Nrf2* through the action of p38 MAPL, PKC, and PI3K/AKT cascades. * Induction of antioxidant machinery, especially HO-1 and γ-GCLC.	[69]
Melanoma cells	*Panax ginseng*	Hairy roots derived from cotyledon and hypocotyl cultures	-	* Inhibition of the accumulation of melanin. * Anti-tyrosinase and anti-melanogenic activity of plant extract. * Enhancement of the production of anthocyanin.	[70]
Epidermal melanocyte	*Brassica rapa*	Hairy roots derived from cotyledon culture	-	* Strong inhibitory action against tyrosinase. * Inhibition of melanin production. * Inhibition of AMPc levels. * Repression of the microphtamia-associated transcription factor (MITF)	[71]

**Table 2:** Examples of plant-derived bioactive compounds with antioxidant activity that display outstanding skin anti-ageing properties and have been demonstrated in vitro using murine cell lines.

Cell Line	Plant Species	Plant Material	Bioactive Molecules (If Applicable)	Noticeable Effects	References
Fibroblasts	*Euterpe oleracea* Martius	Fruits	Malvidin and cyanidin derivatives	* Prevention of lipid peroxidation * Reduction in the adverse effects of UVA-induced stress. * Interference in ROS generation. * Maintenance of GSH level at normal.	[72]
	*Nicotiana tabaccum*	Cell suspension derived from leaf segment culture	-	* Low ROS production. * Strong antioxidant capacity of the extract. * Upregulation of genes involved in DNA protection such as *GADD45*, *SIRT-1*, and *SIRT-6*. * Repression of the expression of *MMP* genes.	[73]
	*Thymus vulgaris*	In vivo plant material	-	* Prevention of collagen degradation through the inhibition of *MMP-1* expression. * Inhibition of ROS accumulation. * Increase in DLD production, known as a cellular defense against oxidative stress. * Inhibition of MAPKs and AP-1 signaling pathways.	[74]
	*Solanum lycopersicum*	Cell suspensions derived from leaf culture	Rutin, chlorogenic acid, coumaric acid, ferulic acid, and glucosides	* Lower production of ROS. * Strong expression of antioxidant genes. * Reduction in nuclear DNA damage. * Decrease in the expression of *MMP* genes.	[75]
Keratinocytes	*Solanum lycopersicum*	Cell suspensions derived from leaf culture	Rutin, chlorogenic acid, coumaric acid, ferulic acid, and glucosides	* Lower production of ROS. * Strong expression of antioxidant genes. * Reduction in nuclear DNA damage. * Decrease in the expression of *MMP* genes.	[75]
Melanoma cells	*Brassica rapa*	Hairy roots derived from cotyledon culture	-	* Strong inhibitory action against tyrosinase. * Inhibition of melanin production. * Inhibition of AMPc levels. * Repression of the microphthalmia-associated transcription factor (MITF).	[71]

**Table 3:** Overview of the main elicitors that have been evaluated to enhance the production of desired PDBCs in plant cell culture (PCC) systems.

Elicitor Type (Abiotic/Biotic/Combined)	Elicitors	Culture Type	Plant Species	Bioactive Compounds Identified in the PCC System	References
**Abiotic**	Salicylic acid	Cell suspensions	*Orostachys cartilaginous*	quercetin, quercetin-3-O-glucose kaempferide, kaempferol-3-rutinoside, and epicatechin gallate	[116]
	Salicylic acid	Cell suspensions	*Pueraria candollei*	Khwakhurin, daidzein, puerarin, and genistin	[57]
	Salicylic acid	Cell suspensions	*Phoenix dactylifera* L.	Catechin, apigenin, kaempferol, caffeic acid	[117]
	Salicylic acid and Methyl jasmonate	Cell suspensions	*Thevetia peruviana*	N/A	[118]
	Methyl jasmonate	Hairy roots	*Glycyrrhiza inflata*	Glycyrrhizin	[119]
	Methyl jasmonate and titanium oxide nanoparticles	Callus	*Salvia tebesana*	O-diphenols, avonol phenolic acid, avonoid, and avone	[120]
	Methyl jasmonate and cyclodextrin	Hairy roots	*Arachis hypogea*	Stilbene	[121]
	Jasmonic acid and sucrose	In vitro shoot culture	*Musa* sp.	N/A	[122]
	Zinc oxide nanoparticles	Callus	*T. vulgaris*, *T.daenensis*, *T. kotschyanus* and *Zataria multiflora*	Thymol and carvacrol	[123]
	Silver nanoparticles	Callus	*Caralluma tuberculata*	N/A	[124]
	Silver nitrate and silver nanoparticles	Hairy roots	*Cucumis anguria* L.	Coumaric, p-coumaric, vanillic acid, ferulic, caffeic, protocathechuicand t-cinnamic	[125]
	Copper oxide nanoparticles	In vitro shoot culture	*Stevia rebaudiana*	Steviol glycosides	[126]
	Titanium dioxide nanoparticles and silicon dioxide	Callus	*Argania spinosa*	α-tocopherol	[127]
	Silicon oxide	Callus	*Ammi majus*	umbelliferone	[128]
	Silicon oxide	Hairy roots	*Ammi majus*	Umbelliferone	[128]
	Gold, silver, and NAA	Callus	*Prunella vulgaris*	N/A	[129]
	NaCl	Callus	*Cynara cardunculus*	N/A	[114]
	Blue light	Callus	*Solanum xanthocarpum*	ethyl-caffeate, esculetin, caffeic acid, and scopoletin	[113]
**Biotic**	Yeast extract	Hairy roots	*Salvia miltiorrhiza*	Diterpenoid tanshinones	[130]
	*Bacillus cereus*, *Staphylococcus aureus*	Hairy roots	*Datura metel*	Scopolamine	[131]
**Combined**	Methyl jasmonate and chitosan	Cell suspensions	*Pueraria candollei*	khwakhurin, daidzein, puerarin, and genistin	[57]
	Methyl jasmonate and yeast extract	Cell suspensions	*Panax ginseng*	Saponin	[132]
	Yeast extract & Ag^+^, Pb^2+^ and Cd^2+^ elicitors	Cell suspensions	*Linum album*	Podophyllotoxin	[133]
	Methyl jasmonate, chitosan, and vanadyl sulfate	Hairy roots	*Panax ginseng*	ginsenoside	[134]

## Data Availability

Data sharing not applicable to this article as no data sets were generated or analyzed during the present study.
